# Tobacco Hornworm (*Manduca sexta*) caterpillars as a novel host model for the study of fungal virulence and drug efficacy

**DOI:** 10.1080/21505594.2020.1806665

**Published:** 2020-08-25

**Authors:** Naomi Lyons, Isabel Softley, Andrew Balfour, Carolyn Williamson, Heath E. O’Brien, Amol C. Shetty, Vincent M. Bruno, Stephanie Diezmann

**Affiliations:** a School of Molecular Cell Biology and Biotechnology, Tel Aviv University, Tel Aviv, Israel; b Department of Biology & Biochemistry, University of Bath, Bath, UK; c MRC Centre for Neuropsychiatric Genetics & Genomics, Division of Psychological Medicine & Clinical Neurosciences, Cardiff University, Cardiff, UK; d Institute for Genome Sciences, University of Maryland School of Medicine, Baltimore, MD, USA; e School of Cellular and Molecular Medicine, University of Bristol, Bristol, UK

**Keywords:** *Manduca sexta*, caterpillar, fungal virulence, *Candida*, *Cryptococcus*, *Saccharomyces*, *Metschnikowia*, antifungal drug, host model, fungal burden

## Abstract

The two leading yeast pathogens of humans, *Candida albicans* and *Cryptococcus neoformans*, cause systemic infections in >1.4 million patients worldwide with mortality rates approaching 75%. It is thus imperative to study fungal virulence mechanisms, efficacy of antifungal drugs, and host response pathways. While this is commonly done in mammalian models, which are afflicted by ethical and practical concerns, invertebrate models, such as wax moth larvae and nematodes have been introduced over the last two decades. To complement existing invertebrate host models, we developed fifth instar caterpillars of the Tobacco Hornworm moth *Manduca sexta* as a novel host model. These caterpillars can be maintained at 37°C, are suitable for injections with defined amounts of yeast cells, and are susceptible to the most threatening yeast pathogens, including *C. albicans, C. neoformans, C. auris*, and *C. glabrata*. Importantly, fungal burden can be assessed daily throughout the course of infection in a single caterpillar’s feces and hemolymph. Infected caterpillars can be rescued by treatment with antifungal drugs. Notably, these animals are large enough for weight to provide a reliable and reproducible measure of fungal disease and to facilitate host tissue-specific expression analyses. *M. sexta* caterpillars combine a suite of parameters that make them suitable for the study of fungal virulence.

## Introduction

Fungal infections pose a serious threat to human health and well-being worldwide. Each year, as many, if not more patients, die of fungal infections than of malaria or tuberculosis [1]. The leading yeast pathogens, *Candida albicans* and *Cryptococcus neoformans*, account for ~1,400,000 life-threatening infections worldwide with mortality rates of up to 70% [1,2]. Candidemia, most frequently caused by *C. albicans*, is the fourth most common cause of nosocomial blood stream infections, only surpassed by infections with Staphylococci and *Enterococcus* spp. Disturbingly, candidemia incidence rates are on the rise. Within less than ten years, they increased by 36% [3]. Although cryptococcosis incidence rates are on the decline in North America, cryptococcosis as an AIDS-defining illness is responsible for 15% of all AIDS-related deaths worldwide [3,4]. This dire situation is further confounded by the emergence of drug-resistant yeast species, such as *C. glabrata* and *C. auris*. Patients at risk of developing invasive candidemia are often prophylactically treated with fluconazole and echinocandins as a first line defense strategy [5]. Yet, *C. glabrata*, the most common non-*albicans Candida* species associated with nosocomial blood stream infections [6], is intrinsically less susceptible to azole drugs and acquires resistance to echinocandins rapidly [7]. The rapid global spread of multidrug-resistant *C. auris* has further exacerbated the threat posed by fungal infections. *C. auris* was first reported in 2009 in Japan [8]. In 2015, *C. auris* arrived in Europe causing an outbreak involving 72 patients in a cardio-thoracic hospital in London [9]. *C. auris* outbreaks have been reported from South Korea, India, Spain, Columbia, Switzerland, Germany, Israel, Kuwait, and Oman [10]. Most concerningly, up to 25% of *C. auris* isolates are multidrug resistant, with some strains being resistant to three of the four drug classes available for the treatment of systemic candidemia. In addition to the unacceptably high burden on human health, fungal infections substantially increase health care costs. Treatment of candidemia requires extended hospitalization, resulting in additional costs of up to 45,000 USD in adult patients or up to 119,000 USD in pediatric patients [11].

It is thus imperative to investigate fungal virulence and host response mechanisms. This is traditionally done in mammalian models. Mice are by far the most frequently deployed host model. The tail vein infection model of candidemia, the gastrointestinal infection model of candidemia, the *Candida* vaginitis model [12] and the mouse inhalation model of cryptococcosis [13] are used extensively. While mice outnumber rabbits, specific aspects of fungal disease, such as chronic cryptococcal meningitis [14], and *Candida* keratitis [15] are often studied in rabbits. Both mammalian models combine a number of features that make them particularly amenable for the study of fungal diseases, such as susceptibility, availability of knockout mutants, and histology comparable to human disease. Yet, using mammals is ethically controversial, costly, and requires extensive board certifications and documentation.

In an effort to reduce the usage of mammalian hosts, alternative invertebrate models have been developed and successfully used over the past two decades. The most commonly employed invertebrate species include the nematode *Caenorhabditis elegans*, the fly *Drosophila melanogaster*, and larvae of the Greater Wax moth *Galleria mellonella*. All three can be easily maintained in the laboratory, are much less expensive than mice or rabbits, and have been used for the study of diverse yeast pathogens, such as *C. neoformans* [16,17], *C. albicans* [17–19], *C. parapsilosis* [19–21], *C. glabrata* [20,22]. Of note, invertebrate models differ in their applicability and the best suitable model should be carefully selected [23]. Unlike mammalian models, these invertebrates do not have adaptive immunity but all share components of the innate immune system [24,25], some of which are conserved with mammals. This includes the Toll-like receptors found in the fly [26] and the homolog of the MKK3/6 kinase in the nematode [27]. Ironically, it is the Toll-like receptors that protect flies from infections with *C. neoformans* [28], *C. albicans* [29], and *C. glabrata* [30] and the MKK3/6 homolog SEK-1 protects the nematode from bacterial invaders [27]. Thus, to increase susceptibility of flies and nematodes to fungal pathogens, Toll and *sek-1* [31] mutants are required. A key limitation for the study of human pathogens, is the inability of nematodes and flies to survive human body temperature. Only *Galleria* can withstand 37°C [17]. Due to their long-standing history as eukaryotic models, well-curated genomes and genome databases exist for the nematode and the fly. Although the *Galleria* genome has been announced recently [32], detailed analyses and annotations are still outstanding. Delivery of an exact inoculum of fungal cells is crucial when comparing mutant and wild-type yeast strains, neither fly nor nematode permit routine direct injection. *Galleria* can be directly injected with a defined cell number. Extensive stock collections provide fly and nematode strains, while until recently, *Galleria* larvae had to be purchased from fishing shops. Now, UK-based TruLarv is selling research grade larvae.

Tobacco Hornworms are most common in the southern United States, where they feed on solanaceous plants and are thus considered a plant pest. As an insect model with a long history in research, *Manduca sexta* has yielded important insights into flight mechanisms, nicotine resistance, hormonal regulation of development, metamorphosis, antimicrobial defenses, and bacterial pathogenesis. *M. sexta* laboratory stocks have been derived from animals collected in North Carolina, USA [33] and have been maintained in laboratories on both sides of the Atlantic for several decades. *M. sexta*’s research portfolio includes a draft genome sequence that has been complemented with tissue-specific transcriptomic analyses [34], numerous successful applications of RNAi [35–39]. Down-stream analyses [40] of the animal’s innate immunity [41], which also comprises b-(1,3)-glucan recognition proteins [41], are facilitated by protocols for the efficient extraction of hemocytes. Despite its versatility, *M. sexta* has yet to be explored for its suitability as a host model for fungal infections.

Here, we aimed to establish *M. sexta* as a novel model host for the study of fungal virulence. Inbred animals from the University of Bath’s research colony were tested for their ability to live at 37°C, their susceptibility to different yeast species, and the reproducibility of *C. albicans* mutant phenotypes obtained in mice virulence studies. Indeed, the caterpillars grow at 37°C while maintaining susceptibility to the leading yeast pathogens *C. albicans, C. neoformans*, and the emergent *C. auris*. Specific *C. albicans* mutants are just as attenuated in their virulence in *M. sexta* as they are in mice. To expand *M. sexta’s* applicability as a host model, we developed an infection protocol that permits screening of fungal burden throughout the course of infection in a single animal and uses weight as a proxy measure for virulence in addition to survival. *M. sexta* can furthermore be used to test efficacy of common antifungal drugs and to query the host transcriptional response to systemic yeast infections. Our results define *M. sexta* characteristics that recommend these caterpillars as a nonmammalian host model for the study of fungal virulence with susceptibility to different yeast species.

## Materials and methods

### Origin of the Bath colony of Manduca sexta

The University of Bath colony has been in continuous culture since 1978 without the addition of animals from elsewhere. Bath’s genetic stock was derived from animals from the Truman-Riddiford laboratories at the University of Washington in Seattle, USA. Their animals date back to the ones originally collected in North Carolina in 1976 [33]. Researchers in Europe wishing to obtain *M. sexta* caterpillars, please contact manduca@bath.ac.uk. Researchers in North America can purchase *M. sexta* caterpillars from the Carolina Biological Supply Company (item #143,882).

### Caterpillar maintenance and yeast culture conditions

*M. sexta* caterpillars from Bath’s facility were reared to fifth instar under standardized conditions. They were maintained in 125 ml disposable cups (Sarstedt Ltd., Cat. No. 75.1335) on a wheat germ-based diet (Table S1) at a constant temperature of 25°C with 50% humidity and 12 hours light/dark cycles. Three days prior to fungal inoculations, animals were shifted to a formaldehyde-free diet as formaldehyde is toxic to nonmethylotrophic yeast.

For infection assays, yeasts were grown overnight in 50 ml YPD (1% yeast extract, 2% peptone, 2% dextrose) at 30°C and cells were harvested by centrifugation for 3 minutes at 3,000 rpm. The cell pellet was washed twice with 1x phosphate buffered saline (PBS) and suspended in 5 ml 1x PBS. Cells were counted and numbers adjusted as indicated. *C. albicans* YSD85 (Table 1) cells were heat-inactivated by incubation at 65°C for 20 minutes. For long-term storage, yeasts were cryo-archived at −80°C in 25% glycerol.

**Table 1.** Yeast strains used in this study.

Strain ID	Strain Name (genotype)	Species	Source
YSD89	SN95	*C. albicans*	63
YSD85	SC5314	*C. albicans*	
YSD87	BWP17	*C. albicans*	42
YSD190	VIC84 (*cka2∆/∆*)	*C. albicans*	43
YSD192	VIC93 (*cka2/cka2::CKA2*)	*C. albicans*	43
YSD302	CAS12 (*ahr1∆/∆*)	*C. albicans*	65
YSD303	CAS13 (*ahr1/ahr1::AHR1*)	*C. albicans*	65
YSD304	RM1000	*C. albicans*	44
YSD305	JC52 (*hog1/hog1::HOG1*)	*C. albicans*	44
YSD306	JC50 (*hog1∆/∆*)	*C. albicans*	44
YSD883	*hog1∆/∆*	*C. albicans*	This study
YSD790	YPS143	*S. cerevisiae*	45
YSD1448	NCYC2580	*M. pulcherrima*	46
YSD465	2001	*C. glabrata*	47
YSD1454	TA004-14	*C. auris*	48
YSD1028	H99	*C. neoformans*	49

### Yeast infections and measurements of fungal burden and drug efficacy

100 µl of washed and number-adjusted yeast suspension were injected into each caterpillar’s distal right proleg with a 30G1/2” needle (BD Microlance) and a 1 ml NORM-JECT syringe. Following injection, each animal’s weight was recorded. Animals were scored for survival and weight once daily for three or four days post infection. During the course of the experiment, animals were kept on their regular diet (Table S1), on a 12-hour light/dark cycle at the temperature indicated.

To measure fungal burden in caterpillar feces and hemolymph, six animals were injected with either 100 µl of 1x PBS or 10^6^ cells of the wild type SN95 (YSD89) or the *hog1∆/∆* mutant strain YSD883 (Table 1) and kept at 37°C. On infection day (Day 0), feces and hemolymph were dissolved in 1x PBS and spread without further dilution onto YPD. On Day 1, two animals were selected from each group and their hemolymph and feces were collected daily throughout the course of infection (Days 1–3). To collect hemolymph, animals were first kept on ice for 15 minutes. The “horn” was then surface sterilized with 70% ethanol and its top 1–2 mm clipped with a pair of micro scissors. Hemolymph was collected in a prechilled 1.5 ml Eppendorf tube and cooled immediately to reduce polymerization and melanization. One fecal pellet was collected daily with sterile forceps, weighted and suspended in 500 µl 1x PBS. Prior to diluting, the suspension was thoroughly vortexed for 10 seconds, and centrifuged for 5 seconds using a table-top centrifuge to separate fecal matter. To quantify fungal burden, hemolymph and fecal samples were plated either directly onto YPD-agar with 50 µg/ml Kanamycin or in ten-fold serial dilutions. Agar plates were incubated at 30°C for 48 hours and colonies counted.

To assess the efficacy of commonly used antifungal drugs, animals were infected with 10^7^ cells of YSD85 (Table 1) or 100 µl PBS and treated with increasing doses of fluconazole and caspofungin (Sigma Aldrich, Inc.) as indicated. Drugs were injected 30 minutes postinfection with an ethanol-sterilized Hamilton syringe in a total volume of 10 µl per animal. Caterpillars were weighed and scored for survival on the day of injection and the following three days.

### Statistical analyses

Kaplan-Meier estimators were calculated from the survival data using the Surv function in the “survival” R package [50], implementing right censoring for surviving animals. Survival curves were plotted using the “survminer” R package [51]. Differences between treatments were evaluated with a log rank test comparing either different yeast inoculum sizes to each other or different drug concentrations to the vehicle only controls. Weight and fungal burden were plotted using ggplot2 [52] and weight differences were evaluated using linear models with day postinoculation and the interaction between treatment and days post infection as fixed effects and individual as a random effect using the lme function from the “nlme” R package [53]. The survival and weight analyses code can be found here: https://github.com/hobrien/DiezmannLabManduca/blob/master/R/Results.Rmd. All analyses were conducted in R version 3.6.0.

### RNA sequencing and gene expression analysis

Five animals were injected with 100 µl 1x PBS or 10^6^ yeast cells of the wild-type strain SN95 (YSD89) (Table 1) and maintained on a 12-hour light/dark cycle at 37°C for 24 hours. Three animals were then randomly selected for transcriptomic analyses of their midguts. In preparation for midgut extraction, animals were surface sterilized and midgut tissue extracted and washed as described before [34]. Midgut RNA was extracted using TRIzol reagent (Invitrogen, Inc.), quantified using the Agilent 2100 Bioanalyzer system and used to generate RNA-seq libraries (strand-specific, paired end) using the TruSeq RNA sample prep kit (Illumina).

150 nucleotides of the sequence were determined from both ends of each cDNA fragment using the HiSeq 2500 platform (Illumina). An average of 83.79 million read pairs were obtained for each sample (range: 51.4 M to 109.8 M read pairs). Sequencing reads were aligned to a combination of the *Manduca sexta* whole genome assembly v1.0 (https://i5k.nal.usda.gov/Manduca_sexta) and the *Candida albicans SC5314* genome version 4 (http://fungi.ensembl.org/Candida_albicans_sc5314_gca_000784635) using HISAT2 [54]. HTSeq [55] was used to generate read counts for each gene. RNAseq data are available at NCBI’s Short Read Archive (PRJNA629104).

Statistical analysis of differential gene expression was performed using the DEseq2 package from Bioconductor [56] and the SARTools pipeline [57]. A gene was considered differentially expressed if the FDR-value for differential expression was less than 0.10. A heatmap of all differentially expressed genes was made using the heatmap.2 function from the “gplots” R package [58].

In order to compare the *M. sexta* transcriptional response to *C. albicans* infection by to that of a mouse, we generated a blast reference database using the peptide sequences for 67,960 proteins from the mouse genome (GRCm38) available from Ensembl (https://useast.ensembl.org). We downloaded the peptide sequences for 27,403 proteins from the *M. sexta* database [34]. We computed peptide sequence similarity between the *M. sexta* proteins and the mouse proteins using the blastp local alignment search tool (ncbi-blast+ v2.8.1) [59]. For each *M. sexta* protein, we had multiple hits that were ranked based on the e-value computed by the blastp search tool. For the list of differentially expressed *M. sexta* genes from each comparison, we extracted the mouse orthologs with e-values smaller than 10^−20^. This list of orthologs was then compared to differential expression lists based on RNA-seq analysis of kidneys [60], tongues [61], and vaginas [62] from *C. albicans* infected mice. Gene lists were compared using VennDiagram (https://cran.r-project.org/web/packages/VennDiagram/VennDiagram.pdf).

## Results

### M. sexta caterpillars are susceptible to the leading fungal pathogen C. albicans at 37°C

**Figure 1.**
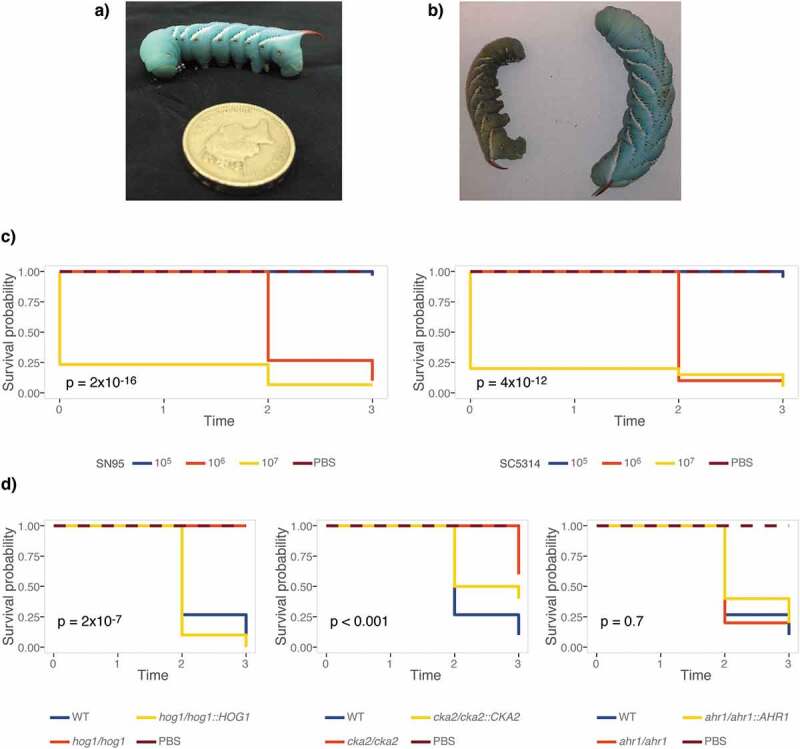
*M. sexta* caterpillars are susceptible to infections with *C. albicans* at their standard maintenance temperature of 25°C. (a) 13-day old *M. sexta* fifth instar caterpillar prior to injection, weighing ~2 g. Its distinguishing feature, the reddish horn at the posterior, is clearly visible. (b) 24 hours post injection with the *C. albicans* wild-type strain SC5314, the dead animal on the left has lost color and turgidity compared to the live PBS control animal on the right. (c) Groups of 10 animals were infected with *C. albicans* SN95 or SC5314 wild-type strains. Kaplan-Meier curves show dose-dependent killing of caterpillars as indicated by the inset p-values for overall differences, excluding PBS controls. (d) Survival curves of 10 animals per group infected with 10^7^ cells of *C. albicans* mutants that have attenuated virulence phenotypes in mice or epithelial cell models. The *hog1∆/∆* and *cka2∆/∆* mutants exhibit attenuated virulence, while virulence of the *ahr1∆/∆* mutant is comparable to wild type. The *hog1∆/∆* mutant differs significantly from the wild type (p = 0.0001), while the complemented strain *hog1/hog1::HOG1* kills *M. sexta* at a level comparable to that of the wild-type strain (p = 0.21). The *cka2∆/∆* mutant is significantly less virulent than the wild type (p = 0.00021), while the complemented strain *cka2/cka2::CKA2* is not (p = 0.054). Shown is one of two comparable biological replicates.

We first aimed to determine if *M. sexta* fifth instar caterpillars (Figure 1(a)), reared and maintained at standard conditions, are susceptible to *C. albicans*. To do so, groups of ten animals were infected with increasing doses of the widely used *C. albicans* laboratory strains SC5314 and SN95 [63] (Table 1). Animals were kept at 25°C, their standard maintenance temperature, and scored for survival on three consecutive days. Dead animals go limp and turn brown-green in color, which is in stark contrast to the vivid turquoise of live animals (Figure 1(b)). Caterpillars infected with *C. albicans* succumbed in a dose-dependent manner. Both *C. albicans* strains killed *M. sexta* efficiently at inocula of 10^6^ or 10^7^ cells per animal (Figure 1(c)). To assess if survival measures in caterpillars are comparable to those obtained in the current gold standard, the murine model of systemic candidemia, we tested *C. albicans* mutants with published phenotypes of either attenuated virulence, such as the *hog1∆/∆*[64] and *ahr1∆/∆*[65] mutants, or wild-type levels of virulence, such as *cka2∆/∆*[66] (Table 1). Hog1 is just as essential for virulence in caterpillars as it is in mice. Cka2 is not required to establish systemic infections in mammals but is in caterpillars. Ahr1, while required for virulence in mammals, appears to be dispensable for virulence in caterpillars (Figure 1(d)).

**Figure 2.**
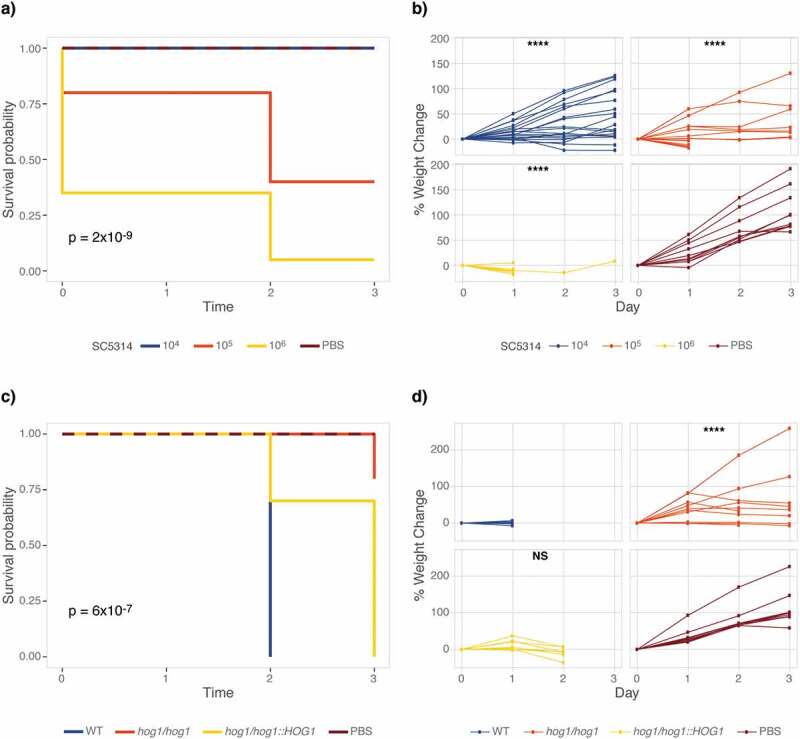
Elevated temperature increases susceptibility of *M. sexta* to *C. albicans*. Groups of 10 animals were injected with increasing doses of the *C. albicans* wild-type strain SC5314 or with 10^6^ cells of the *hog1∆/∆* mutant strain and its complemented control and wild-type progenitor and kept at 37°C for the duration of the experiment. (a) *M. sexta* caterpillars succumb to infection with 10^5^ cells of the laboratory strain SC5314 at 37°C. (b) Weight gain of caterpillars infected with *C. albicans* was significantly reduced at all doses relative to PBS controls (**** = P < 0.0001). (c) Attenuated virulence of the *hog1∆/∆* mutant is retained at 37°C. (d) Animals infected with the *hog1∆/∆* mutant gain significantly more weight than those infected with the wild-type strain RM1000 (**** = P < 0.0001). Infection with the *hog1/hog1::HOG1* complemented strain resulted in a lack of weight gain comparable to wild type (p = 0.16). Shown is one of two comparable replicates.

Given the importance of temperature for fungal virulence, we investigated if *M. sexta* retained their susceptibility to *C. albicans* at human body temperature. We first aimed to establish the effect of raising the temperature from 25°C to 37°C on uninfected caterpillars’ survival and development. All animals survive four-day exposure to 37°C but grow slower than at 25°C (p = 0.0001) (Fig. S1). Next, we quantified survival of animals infected with *C. albicans* wild type at 37°C (Figure 2(a)). 10^5^ *C. albicans* cells elicit 50% mortality on Day 2, while 10^6^ *C. albicans* cells lead to 100% mortality on Day 3. In comparison, 10^7^ cells are required for the same outcome at 25°C (Figure 1(b)). Increased susceptibility at 37°C may be due to the temperature-dependent expression of *C. albicans* virulence traits, such as the yeast-to-hyphae transition, which is triggered at 37°C. To exclude the possibility that mortality is due to starvation rather than the outcome of a host-pathogen interaction, we infected caterpillars with live and heat-killed *C. albicans* wild-type cells and measured survival and weight at 37°C. Only live cells, but not heat-killed yeast cells, kill caterpillars suggesting that killing is not due to nutritional limitations (Fig. S2a). Weight gain, however, was larger in caterpillars injected with heat-killed *Candida* cells than PBS, indicating that dead yeast cells may be of nutritional value to *M. sexta* (*p* < 0.0001) (Fig. S2b).

Demonstrating susceptibility of *M. sexta* caterpillars to *C. albicans* lent support to their suitability as an alternative host model for the study of fungal virulence and emphasized the need for additional measures of fungal virulence. To add granularity to fungal virulence data collected from *M. sexta*, we complemented measures of survival with quantifications of weight. To collect weight data, caterpillars were weighed prior to infection and then daily. Weight gain in animals infected with a low dose of 10^4^ cells did not significantly differ from those injected with 1x PBS (*p* = 0.5847) but caterpillars infected with10^5^ cells per animal exhibited significant weight loss (*p* = 0.0001). Too few animals survived infection with 10^6^ cells to allow for a meaningful comparison (Figure 2(b)). After establishing susceptibility of *M. sexta* to *C. albicans* at 37°C, we aimed to validate the attenuated virulence phenotype of the *hog1∆/∆* mutant strain. To test this, caterpillars were infected with 10^6^ cells of the wild-type strain, the *hog1∆/∆* deletion mutant, and the *hog1/hog1::HOG1* complementation strain (Table 1). Virulence in the *hog1∆/∆* mutant is significantly attenuated at 37°C (Figure 2(c)). Weight gain was larger in animals infected with the *hog1∆/∆* mutant strain than those infected with the wild-type strain (*p* < 0.0001), while infection with the complemented strain lead to a comparable lack in weight gain as in wild type infected animals (Figure 2(d)).

### Measurements of fungal burden in feces and hemolymph obtained from a single animal throughout infection provides a useful measure of virulence

**Figure 3.**
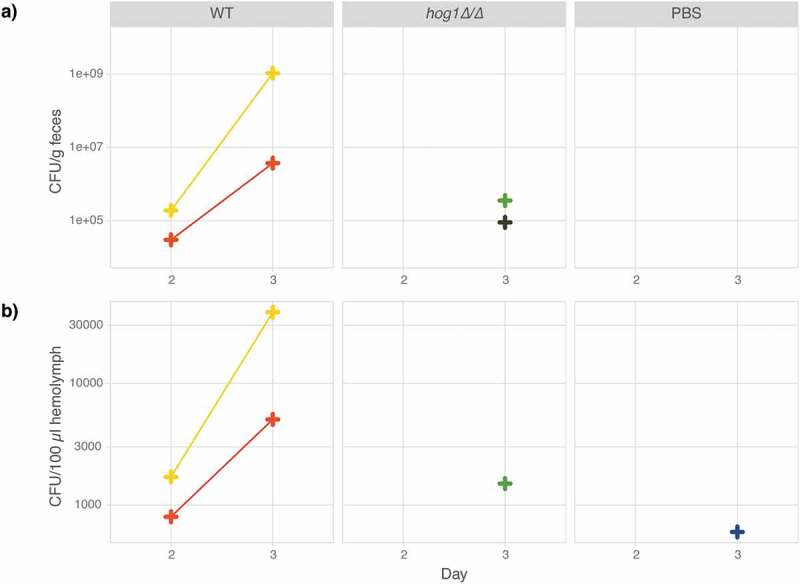
Fungal burden in caterpillar feces and hemolymph as a measure of disease progression. Six animals were injected with either 100 µl 1x PBS or 10^6^ cells of the wild-type strain SN95 or its *hog1∆/∆* derivative (YSD883) and maintained at 37°C. On Day 1, two surviving animals were selected from each group for CFU analysis. Colony forming units per gram feces (a) and 100 µl hemolymph (b) increase in the wild-type strain between Day 2 and 3 but are not detectable in the *hog1∆/∆* mutant nor the control animals until Day 3. Each CFU data point represents the average of at least two (usually three to four) technical replicates.

To further expand the applicability of *M. sexta* caterpillars as a host model for fungal infections, fungal burden in the hemolymph and feces was quantified daily throughout the course of infection. Since the collection of hemolymph or feces does not necessitate killing the animal, data could be collected daily throughout the course of infection for the same caterpillar. Animals infected with the wild type and the *hog1∆/∆* mutant (Table 1) were compared to control animals injected with 1x PBS only and fungal burden measured as colony-forming units (CFUs) in feces and hemolymph in two animals per group. Yeasts were detected in the feces and hemolymph of animals infected with the wild type on Day 2 and CFU counts increased on Day 3 (Figure 3(a,b)). In animals infected with the *hog1∆/∆* mutant, low CFU counts were detected on Day 3 in feces and hemolymph. Even fewer CFUs were detected in the hemolymph of animals injected with PBS on Day 3. Weight measures showed an inverse correlation growth of the pathogen and the host for the *C. albicans* wild-type strain (Fig. S3). Thus, measurements of fungal burden, that can be obtained from the same animal throughout the course of infection, provide a valuable parameter for the study of fungal virulence.

### Caterpillars are suitable for antifungal drug treatment studies

**Figure 4.**
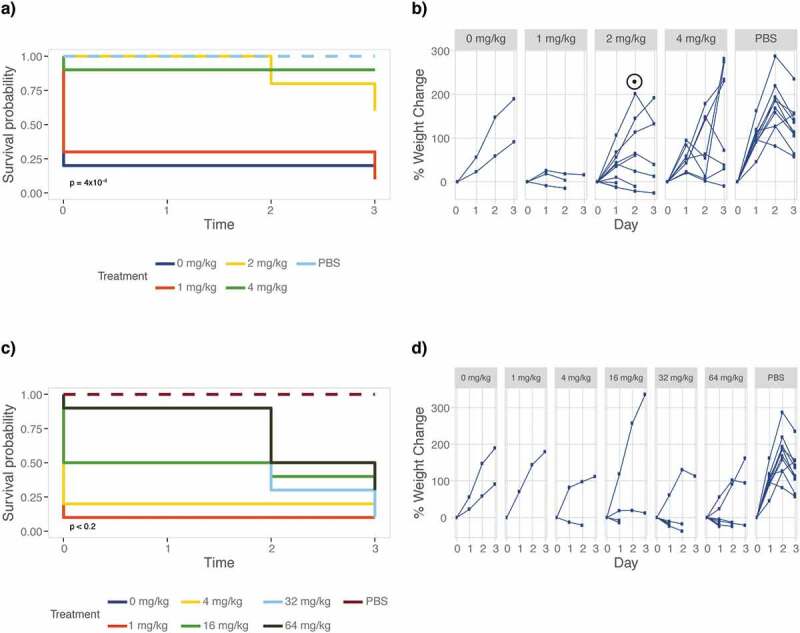
Antifungal efficacy testing of fluconazole and caspofungin. Groups of ten caterpillars infected with 10^6^ cells of *C. albicans* strain SC5314 were treated with increasing doses of antifungal drugs fluconazole or caspofungin and maintained at 37°C for the duration of the experiment. Differences between drug treatments were evaluated using a log rank test comparing animals injected with different amounts of drug to those injected with drug solvent (water or DMSO) only, excluding PBS injection controls. Weight gain was compared to the PBS control due to low survival in the solvent control group. (a) Animals were scored daily for survival showing survival improved significantly in animals receiving caspofungin treatment. Inset *p* value relative to drug solvent control. (b) Rate of weight gain, however, remains low (*p* < 0.0001) for animals that received 2 or 4 mg/kg caspofungin. The peak and drop in weight, marked with a ⊙, is coinciding with the onset of prepupation. This “pupation drop” is due to the animals refraining from food upon entering the early stages of pupation. (c) Fluconazole treatment improves *M. sexta* survival but weight gain remains low compared to PBS control infected animals (*p* < 0.005) for all treatments. (d) Weight data were not analyzed for significance due to the lack of surviving animals in the no treatment group.

The currently available armamentarium of antifungal drugs is limited and the drugs that are available are often lacking in efficacy. Abilities to study drug efficacy and mode of action in a host model are thus pertinent to the development of novel antifungal drugs. To determine *M. sexta*’s suitability for drug efficacy testing, we recorded survival and weight of animals infected with the *C. albicans* wild-type strain SC5314 (Table 1) that were treated with increasing doses of two common antifungals, fluconazole and caspofungin (Figure 4). Treatment with the echinocandin caspofungin significantly improves caterpillar survival (p = 4x10^−4^) and weight gain (p = 0.0047) when compared to untreated animals (Figure 4(a,b)). Doses of 2 mg/kg (p = 0.01) and 4 mg/kg (p = 0.002) are most effective in improving survival. Treating animals with fluconazole improves survival rates but caterpillars still grow slower (Figure 4(c,d)). Thus treatment with antifungals cures caterpillars from fungal disease.

### M. sexta caterpillars are broadly susceptible to different yeast pathogens

**Figure 5.**
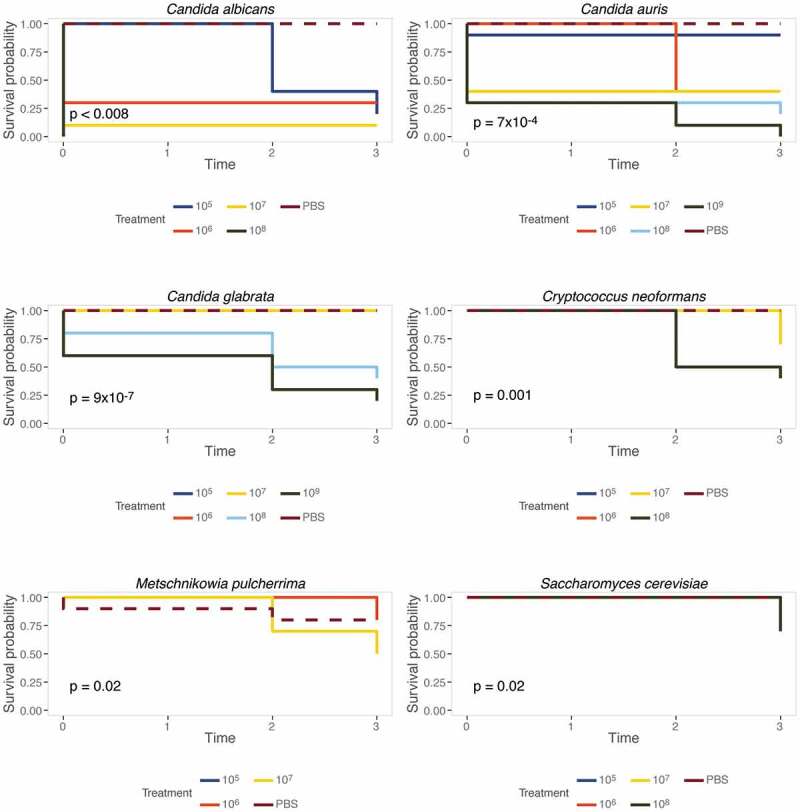
Caterpillars are susceptible to common yeast pathogens. Groups of ten animals were infected with increasing numbers of yeast cells as specified and survival was recorded daily. Inset *p* values represent results from a log rank test of all treatments excluding PBS. Caterpillars are not susceptible to *S. cerevisiae* or *M. pulcherrima* but infections with *C. neoformans, C. glabrata, C. auris,* and *C. albicans* result in significantly reduced survival rates.

**Figure 6.**
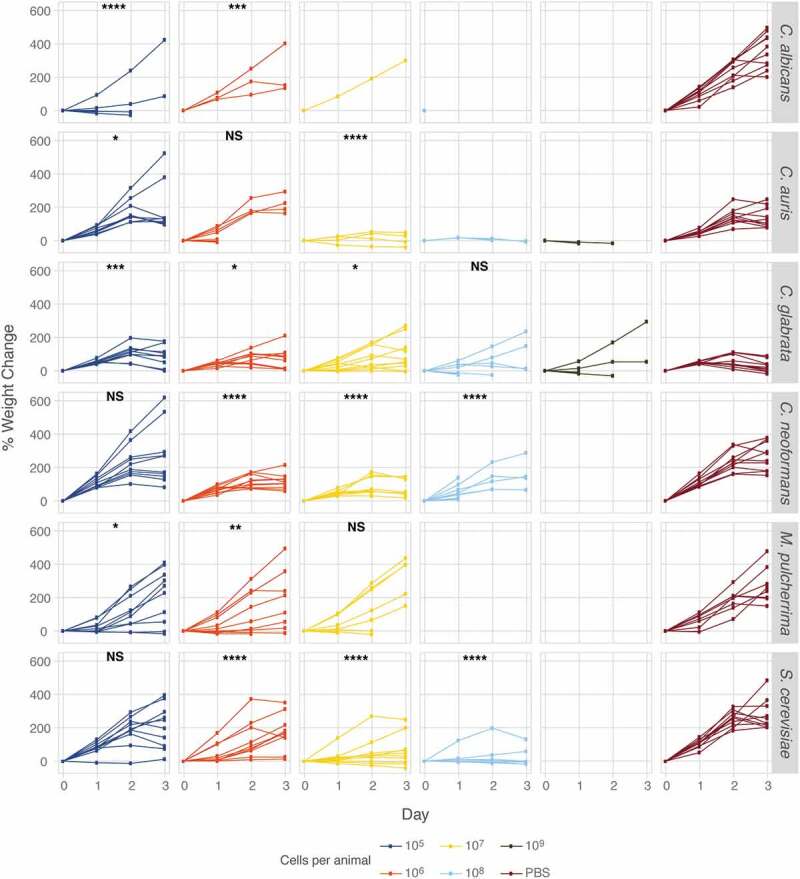
Caterpillar weight as a measure of virulence. Groups of ten animals were infected with increasing amounts of yeast inocula from six different species or PBS and the weight of surviving animals was recorded daily. Weight measures of yeast-infected caterpillars were analyzed for statistical significance revealing that yeast infections affect weight regardless of mortality rates. The slope of each curve at each yeast concentration was compared to the PBS control by fitting a mixed model where caterpillars were treated as a random effect using linear mixed-effects modeling. Statistical significance was assessed as follows: * *p* < 0.05, ** *p* < 0.01, *** *p* < 0.001, **** *p* < 0.0001.

While *C. albicans* is undoubtedly a major fungal pathogen of humans, clinical manifestations of fungal infections are not limited to *C. albicans*. To broaden *M. sexta*’s applicability, we sought to quantify the caterpillars’ susceptibility to other fungal pathogens. To this end, animals were infected with wild-type strains of *C. neoformans, C. auris*, and *C. glabrata* (Table 1). Type strains of *Saccharomyces cerevisiae*, the baker’s or brewer’s yeast and *Metschnikowia pulcherrima* (Table 1), a yeast inhabiting fruits and flowers [67], served as reference points for species with attenuated virulence. Based on our initial *C. albicans* infection assays (Figure 2), animals were infected with increasing doses of yeast cells starting at 10^5^ cells per animal and up to 10^9^ cells per animal and maintained at 37°C. Groups of ten animals per yeast dose and species were then screened for survival and weight daily (Figures 5 and 6). Notably, only infections with pathogenic yeast species affected survival of caterpillars (Figure 5). Infections with *S. cerevisiae* or *M. pulcherrima* barely affected caterpillar survival. Comparing survival amongst the pathogenic yeast species revealed *C. albicans* to be the most severe. 10^7^ *C. albicans* cells result in 100% killing within in one day. 10^9^ *C. auris* cells were required to kill all animals within four days and the same dose of *C. glabrata* resulted in 75% killing. 10^8^ *C. neoformans* cells were required to achieve 50% killing within four days, comparable to *C. glabrata*. It should be noted that due to the high viscosity of the cell suspension, we could not test higher concentrations than the ones stated here. In addition to assessing survival, each surviving caterpillar’s weight was recorded daily.

Interestingly, *S. cerevisiae* and *M. pulcherrima* did not kill caterpillars as effectively as clinical species, yet, animals infected with *S. cerevisiae* did not gain weight as well as uninfected control animals (Figure 6). This is in contrast to animals infected with the *C. albicans hog1∆/∆* mutant whose weight gain was significantly different from wild type infected caterpillars (Figure 2). Infections with *C. albicans* resulted in severely reduced weight gain, even at the lowest yeast dose tested, while only the highest dose of *C. auris* elicited a significant reduction in weight gain. *C. glabrata* infection significantly reduced weight gain at the lowest dose but only slightly at higher doses. Infections with *C. neoformans* resulted in significantly reduced weight gain at all but the lowest concentration tested. Caterpillars displayed variable degrees of susceptibility to different yeast pathogens and with weight providing an additional measure of host damage. Thus, *M. sexta* caterpillars are broadly applicable for the study of fungal virulence.

### M. sexta transcriptional response profiles differ between uninfected and infected animals

As a proof-of-concept we recorded transcriptional profiles in three animals infected with the wild-type strain SN95 (Table 1) and compared them to three uninfected control animals. To do so, we collected the mid-gut, rinsed the tissue thoroughly, and extracted RNA, which was then sequenced using Illumina’ HiSeq 2500 platform. With the vast majority of reads mapping to *M. sexta* rather than *C. albicans* (Fig. S4), animals infected with *Candida* displayed a transcriptional profile different from that of uninfected animals (Fig. S5, Table S2). Infection with the wild type elicited quantifiable transcriptional changes in the caterpillars. 157 *M. sexta* genes were up-regulated and 165 went down when compared to the PBS control animals (Table S2).

**Figure 7.**
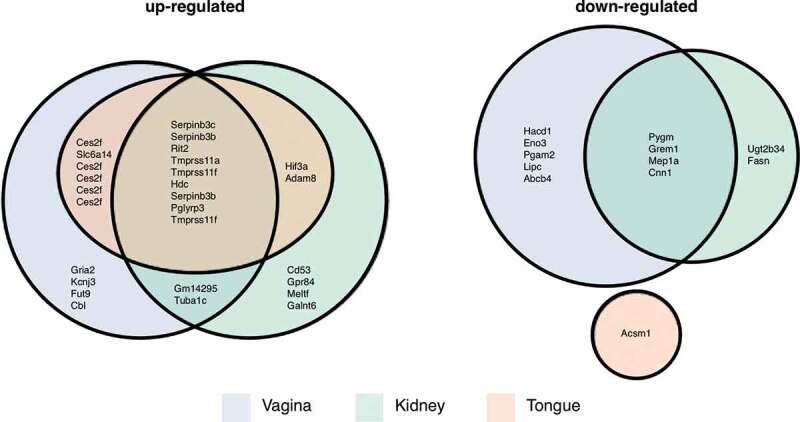
Mouse homologs of *M. sexta* differentially expressed genes. Numbers of *Manduca* genes and their murine homologs that were up- or downregulated in mouse vagina, kidney, or tongue and the overlap between the different data sets as depicted in a Euler diagram. Note, in some cases the same mouse gene was the best BLAST match for several *M. sexta* genes.

Lastly, we compared our set of differentially expressed *M. sexta* genes to those identified in three murine candidemia studies. Here, tissue-specific responses in the kidneys [60], tongues [61], and vaginas [62] were recorded for animals infected with *Candida* wild type (Figure 7, Table S3). *M. sexta* genes up-regulated during infection and their homologs in all three mouse tissues include serine peptidase inhibitors (Serpinb3 c, Serpinb3b), a Ras-like protein (Rit2), transmembrane proteases (Tmprss11a, Tmprss11 f), a histidine decarboxylase (Hdc), and a peptidoglycan recognition protein (Pglyrp3). *M. sexta* genes down-regulated in mouse kidney and vagina include a muscle glycogen phosphorylase (Pygm), a bone morphogenetic antagonist of the DAN family (Grem1), meprin 1 alpha (Mep1a), and calponin 1 (Cnn1). Two of these genes are annotated as functioning in immune system processes (Grem1, Pglyrp3) and three more are relevant for signaling and response to different stimuli (Rit2, Pglyrp3, Pygm). Seven genes, however, are involved in protein metabolic processes (Serpinb3 c, Serpinb3b, Rit2, Tmprss11a, Tmprss11 f, Grem1, Mep1a), suggesting this fundamental process to be of importance in the defense against microbial attacks.

## Discussion

Invertebrate host models have become valuable alternatives to mammalian hosts for the study of fungal disease. Here, we developed *M. sexta* caterpillars as a novel invertebrate model showing that they are naturally susceptible to different human pathogenic yeast species, including the emergent multidrug-resistant *C. auris*. Unlike other host models, these large caterpillars permit daily measures of fungal burden throughout the course of infection in a single animal by either collecting feces or hemolymph. *M. sexta* can be maintained at 37°C, is large enough to be injected with a specified yeast inoculum and for weight to be a reliable measure of virulence. *C. albicans* mutant virulence phenotypes found in mice can be replicated and yeast inocula required to elicit a response in caterpillars are comparable to those used in the murine model. Additionally, the caterpillars permit study of antifungal drug efficacy. While these parameters commend *M. sexta* as a novel host model for the study of fungal virulence, a couple of caveats should be noted. Firstly, as with all animal models, their development follows strict rules. The caterpillars will conclude their fifth instar stadium within 4–5 days, possibly limiting their suitability for some assays. This time span, however, is comparable to the leading insect model, *G. mellonella*, which has successfully been used for antifungal drug evaluations [68], antibacterial testing [69], and testing of gemini surfactants active against *C. albicans* [70]. Secondly, about 10% of infected animals exhibited normal growth and survival. It is currently unclear if this is a limitation of the infection protocol or something to do with the underlying biology of the animals. This will need to be factored in when determining sample sizes for future experiments.

This new model system allows for fungal burden to be monitored throughout the course of infection in a single animal via CFU count. This is unlike any other experimental system, where fungal burden is either an endpoint measure in the mouse kidney, in homogenized wax moth larvae [71], nematodes [72], flies [73], or requires genetically modified fluorescent yeast strains for microscopic imaging and analyses [74]. While we did detect very low CFU counts in the PBS control 3 days post infection, we consider this a spurious finding due to cross contamination as preliminary experiments of plating contents of hemolymph and feces of naïve animals did not detect any yeast growth (data not shown). As a consequence, we amended the protocol to include changing gloves when handling animals of different treatment groups.

When reviewing the weight data collected as part of our study, we noticed that while *S. cerevisiae* has very little effect on caterpillar survival, infections with *S. cerevisiae* led to reduced caterpillar weight gain. A similar, albeit weaker, pattern was observed for infections with the fruit yeast *M. pulcherrima*. The dichotomy between survival and weight observed here further emphasizes that fungal virulence comprises more than a measure of survival. It appears that *M. sexta* would allow discrimination between disease (weight) and death (survival) quantitatively adding further granularity to measuring fungal virulence.

Heat-killed *C. albicans* cells appear to be nonpathogenic in *M. sexta* caterpillars. The lack of mortality in response to inoculation with heat-killed yeast cells indicates that yeast viability and proliferation are required for pathogenesis and excludes the possibility of death due to an allergic reaction in response to a large number of fungal cells. This appears to differ from the responses of other host models to fungal pathogens. While susceptibility was reduced, but still measurable, in *G. mellonella* [75] and the two-spotted cricket [76], heat-killed *C. albicans* cells elicited 100% mortality in a sepsis-like murine model [77]. In mice, serum levels of β-(1,3)-glucan were elevated in animals injected with heat-killed yeasts when compared to those infected with live cells. Indeed, heat inactivation leads to increased exposure of β-(1,3)-glucan on the *C. albicans* cell surface [78] and β-(1,3)-glucan activates the innate immune response in invertebrates and mammals [79]. *M. sexta*’s innate immunity includes a β-(1,3)-glucan recognition protein recognizing *S. cerevisiae*, which is expressed in the fat body and secreted into the hemolymph [80,81] as well as the antifungal molecule diapausin-1, whose expression is developmentally regulated [82]. The lack of response observed here could be due to insufficient expression of either protein or suboptimal exposure of β-(1,3)-glucan in our heat-killed *C. albicans* cells.

Measures of the host transcriptional response provide useful insights into the relevance of specific defense mechanisms. Mouse transcriptional profiles in response to *C. albicans* infections differ between different types of tissues [60–62], which allowed for the identification of tissue-specific response mechanisms such as IL-17 signaling in oral epithelia. Here, we detected numerous differentially expressed genes in the caterpillar midgut when comparing animals infected with *C. albicans* to the PBS control. Given the size of *M. sexta* caterpillars, which permits tissue-specific transcriptional analyses [34], this phenomenon could be systematically investigated in caterpillars rather than mammalian hosts, who are burdened by ethic concerns and economic challenges prohibiting large scale or time-course studies. Furthermore, partial conservation of the transcriptional response to *C. albicans* infections between insects and mammals offers the opportunity to identify and investigate core eukaryotic response mechanisms.

In summary, *Manduca sexta* caterpillars expand the current repertoire of invertebrate models for the study of fungal disease. They combine a suite of measures that commend it as a new model system. Although, *M. sexta* genomic and transcriptomic analyses are currently still in their infancy, we would expect that the Tobacco Hornworm’s long history of being an invaluable model for diverse facets of biology will lead to reliable tools in combination with genetic tractability and protocols establishing the fungus’ fate inside the caterpillar.

## Supplementary Material

Supplemental Material
