# Supplementary material for: Tobacco Hornworm (Manduca sexta) caterpillars as a novel host model for the study of fungal virulence and drug efficacy
Source: Virulence. 2020 Aug 25;11(1):1075–89. doi: 10.1080/21505594.2020.1806665 (PMC7549948; doi:10.1080/21505594.2020.1806665)
Supplement: Supplemental Material [file KVIR_A_1806665_SM0768.zip › Lyons_SOM_Final.docx]

**Supplemental Online Material**

**Figure S1: Caterpillar growth compared between standard colony maintenance temperature and host body temperature.** Groups of ten animals were kept on their standard diet at either 25˚C or 37˚C for four days. All animals were weighted daily as a measure of development. Animals maintained at 37˚C grow slower than those kept at 25˚C (p=0.0001). Shown is one of two comparable replicates.

**Figure S2: Heat-killed *C. albicans* cells do not affect caterpillar survival but contribute to weight gain.** Groups of ten animals were injected with either 10^6^ cells of live or heat-killed *C. albicans* wild-type SN95 (YSD85, Table 1) or PBS as injection control and maintained at 37˚C for the duration of the experiment. **a)** Only live yeast cells kill caterpillars. Heat-killed SN95 cells are avirulent. The inset p-value excludes PBS control. **b)** Animals infected with heat-killed *Candida* cells gain more weight throughout the course of the experiment than animals injected with PBS only (P<0.0001).

**Figure S3: Weight measures of caterpillars infected with *C. albicans* wild-type and *hog1* mutant.** Weights were measured throughout the experiment in parallel to assessment of fungal burden in groups of 2 animals per group. Weight increases in animals infected with *hog1∆/∆* comparable to those injected with PBS, while the animal injected with the *C. albicans* wild type displaying high fungal burden (yellow; Fig. 3) experienced reduced weight gain. The peak and drop in weight observed in the *hog1∆/∆* and PBS animals, marked with a 🞊 is coinciding with the onset of pre-pupation. This ‘pupation drop’ is due to the animals refraining from food upon entering the early stages of pupation.

**Figure S4: Mapping distribution of RNAseq reads.** Between 17.8 M and 34.9 M read pairs per sample mapped either to *M. sexta.* Less than 0.05% of reads mapped to *C. albicans* (none for PBS-injected animals).

**Figure S5: *M. sexta* genomic expression programs in response to infection with *C. albicans*.** Hierarchical clustering of global gene expression patterns recorded in samples from uninfected control animals (PBS, samples A4, D4, E4) and those infected with the *C. albicans* wild type (samples A5, B5, C5). Listed are 322 differentially expressed genes on the y-axis in corresponding order to Table S1. Genes were clustered using complete linkage clustering on Euclidean distances within the expression matrix. In the inset, ‘Count’ refers to the number of differentially expressed genes and value denotes variance stabilizing transformed expression values.

**Table S1: *M. sexta* food mix recipe**

| **Premix** | |  | **Diet cake** | |
| --- | --- | --- | --- | --- |
| Amount | Ingredient |  | Amount | Ingredient |
| 2,700 g | Wheatgerm |  | 336 g | Premix |
| 1,260 g | Casein |  | 1,770 ml | Distilled water |
| 1,080 g | Sucrose |  | 22.5 g | Agar |
| 540 g | Dried active yeast |  | 4 ml | Corn oil |
| 360 g | Wesson’s Salt |  | 4 ml | Linseed oil |
| 36 g | Choline chloride |  | 8 ml | 4% Formaldehyde |
| 72 g | Cholesterol |  | 0.2 g | Chlorotetracycline |
| 36 g | Methyl paraben |  | 0.2 g | Vanderssant vitamins |
| 54 g | Sorbic acid |  | 8 g | Ascorbic acid |

**Food preparation**

For the premix, deactivate the yeast by microwaving for 5 minutes on low power before mixing all components thoroughly. Store in a cool, dry place.

For the diet cake, heat up 650 ml of water on a hot plate while melting the agar in 1 l of water by microwaving. Combine the agar with the pre-warmed water, 336 g of premix, formaldehyde and oils and mix thoroughly using a stand mixer. Dissolve vitamins, antibiotic, and ascorbic acid in the remaining 30 ml of water and add to mixing bowl once the content has cooled below 50˚C to prevent inactivation of vitamins and antibiotic. Line a large ice cube tray with sterile aluminium foil (sterilise by spraying with 70% ethanol) and pour mixture into tray. Let the diet mix set for about 1.5 hours, wrap tightly in aluminium foil and store at 4˚C. Keeps for 3 weeks.
