# Supplementary figures and images for: Tobacco Hornworm (Manduca sexta) caterpillars as a novel host model for the study of fungal virulence and drug efficacy
Source: Virulence. 2020 Aug 25;11(1):1075–89. doi: 10.1080/21505594.2020.1806665 (PMC7549948; doi:10.1080/21505594.2020.1806665)

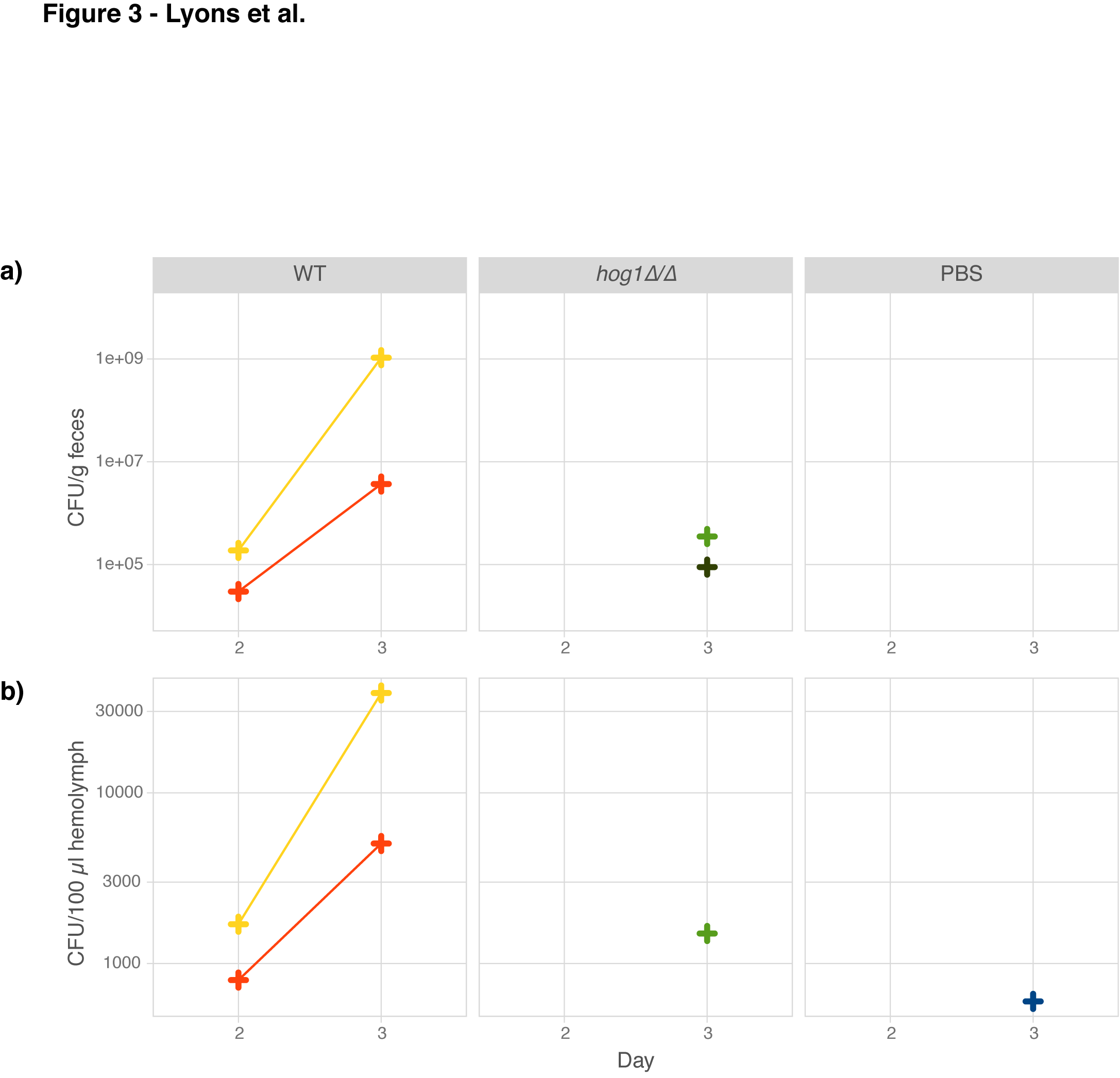

Supplement: Supplemental Material [file KVIR_A_1806665_SM0768.zip › Lyons_Figure_3.tif]

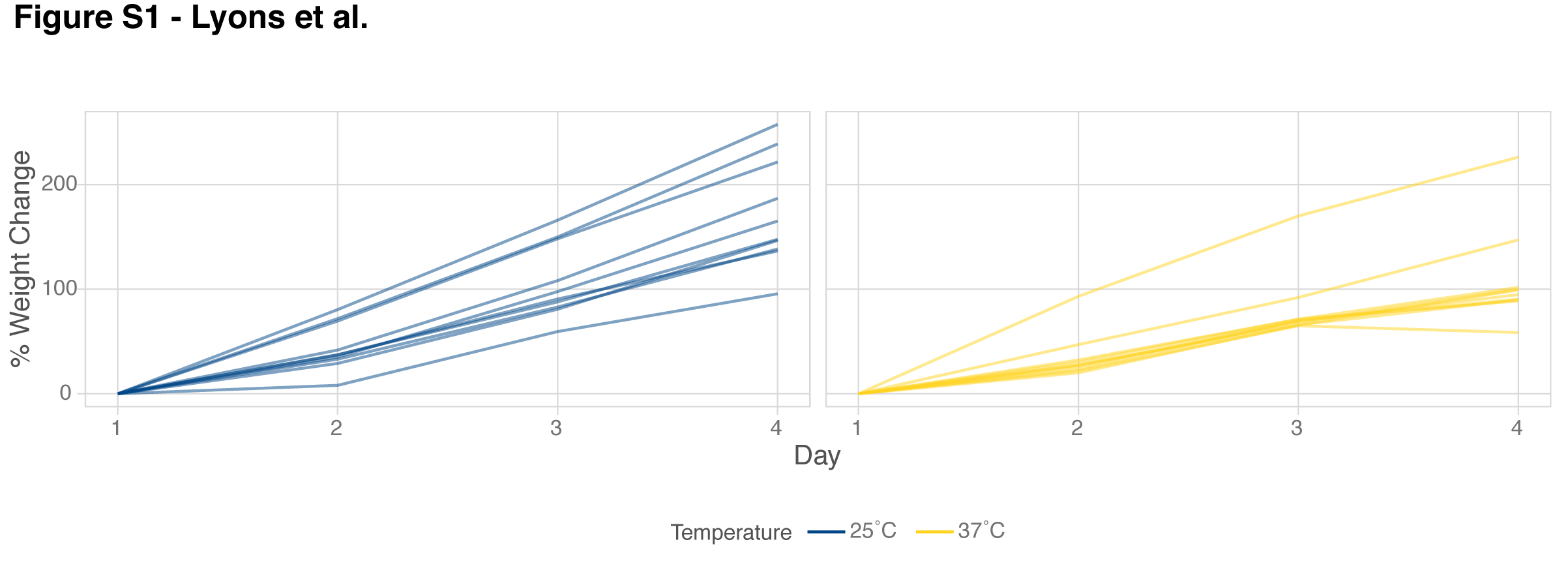

Supplement: Supplemental Material [file KVIR_A_1806665_SM0768.zip › Lyons_Figure_S1.tif]

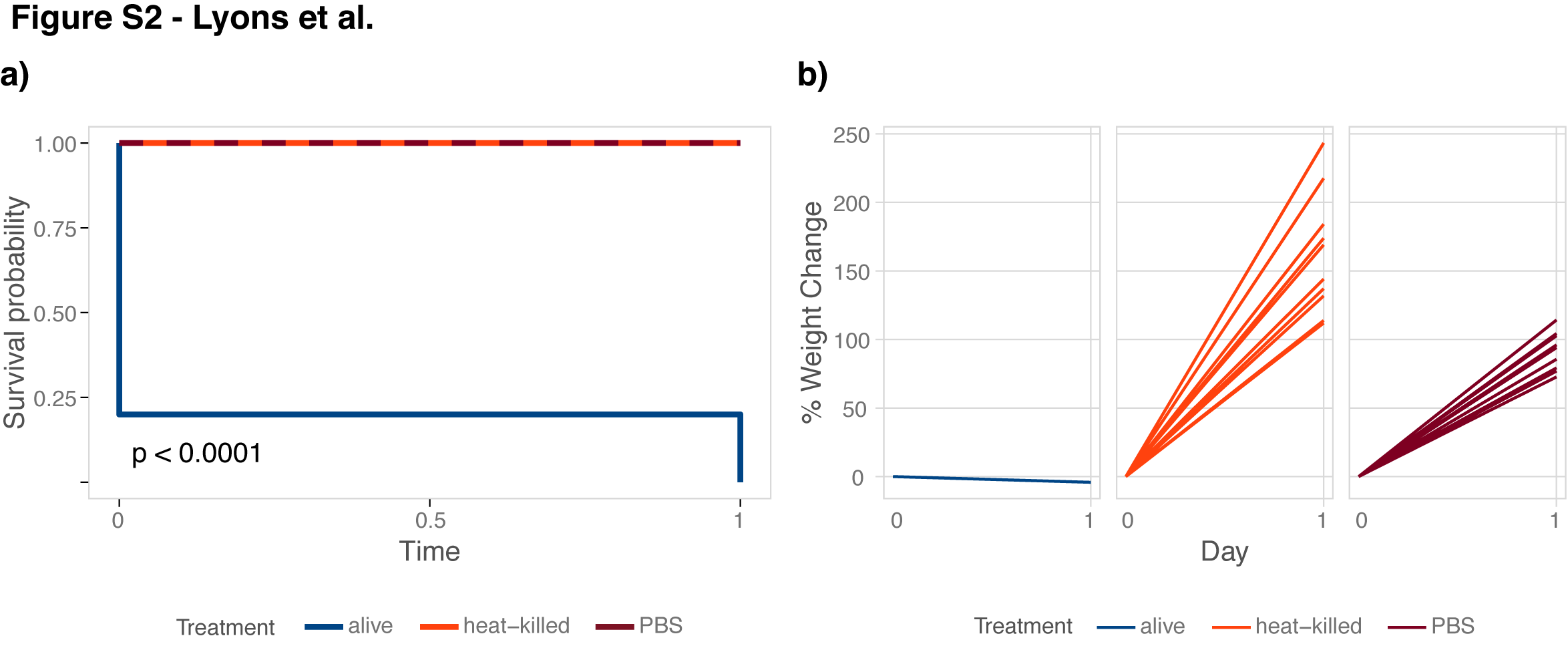

Supplement: Supplemental Material [file KVIR_A_1806665_SM0768.zip › Lyons_Figure_S2.tif]
